# Revisiting the Debate: Does Exercise Build Strong Bones in the Mature and Senescent Skeleton?

**DOI:** 10.3389/fphys.2016.00369

**Published:** 2016-09-13

**Authors:** Julie M. Hughes, Nisha Charkoudian, Jill N. Barnes, Barbara J. Morgan

**Affiliations:** ^1^Military Performance Division, US Army Research Institute of Environmental MedicineNatick, MA, USA; ^2^Thermal and Mountain Medicine Division, US Army Research Institute of Environmental MedicineNatick, MA, USA; ^3^Bruno Balke Biodynamics Laboratory, Department of Kinesiology, University of Wisconsin-MadisonMadison, WI, USA; ^4^John Rankin Laboratory of Pulmonary Medicine, Department of Orthopedics and Rehabilitation, University of Wisconsin-MadisonMadison, WI, USA

**Keywords:** osteogenesis, mechanical loading, aging, sympathetic nervous system, beta-adrenergic receptor, periosteum

## Abstract

Traditional exercise programs seem to be less osteogenic in the mature and post-mature skeleton compared to the young skeleton. This is likely because of the decline in sensitivity of bone to mechanical loading that occurs with advancing age. Another factor contributing to the apparently diminished benefit of exercise in older adults is failure of widely used measurement techniques (i.e., DXA) to identify changes in 3-dimensional bone structure, which are important determinants of bone strength. Moreover, although hormonal contributors to bone loss in the elderly are well-recognized, the influence of age-related increases in sympathetic nervous system activity, which impacts bone metabolism, is rarely considered. In this Perspective, we cite evidence from animal and human studies demonstrating anabolic effects of exercise on bone across the lifespan and we discuss theoretical considerations for designing exercise regimens to optimize bone health. We conclude with suggestions for future research that should help define the osteogenic potential of exercise in older individuals.

In the United States, over 1.5 million osteoporotic fractures occur annually (Black and Rosen, 2016). The majority of these occur in the latter decades of life when rates of bone loss and microarchitectural deterioration are at their greatest (Seeman, 2002). Exercise is a commonly recommended intervention for preventing bone fragility; however, human and animal studies suggest that the anabolic effect of exercise is much less potent in the mature and post-mature vs. the immature skeleton (Forwood and Burr, 1993). These observations raised the question: is exercise a worthwhile strategy for promoting bone health in mature and elderly individuals?

The author of a *New York Times* article (Kolata, 2016) cites minuscule gains in bone density reported from exercise trials in adult populations and concludes that “exercise has little or no effect on bone strength.” This conclusion, however, is based on studies that do not take into account recent advances in non-invasive technologies for measuring bone density and structure (Bouxsein, 2008) or new strategies to make exercise more potent, or osteogenic, in aging populations (Srinivasan et al., 2010).

In this Perspective, we propose that the debate regarding the effectiveness of exercise in promoting bone health in mature and older adults be revisited, focusing on three sometimes overlooked considerations:

1. Appropriate characterization of adaptive responses. Comprehensive assessment of bone structure may be required, in addition to measures of areal bone density, to fully elucidate the impact of exercise.
2. Rethinking of the notion that only high-impact exercise is osteogenic. Innovative exercise paradigms appear to be capable of “hacking” the osteogenic signal produced by exercise such that low-to-moderate intensity activities may also be beneficial.
3. Recognition that the skeleton has strong physiologic interactions with the sympathetic nervous system. Treatments based on exploitation of these interactions may lead to improved bone health in the elderly.

## What is the optimal measure for assessing exercise-induced osteogenesis?

Human studies have demonstrated an age-related decline in the responsiveness of bone mineral density (BMD), as measured by DXA, to exercise interventions (Forwood and Burr, 1993). Nevertheless, a recent meta-analysis in older adults revealed small but statistically significant increases in BMD at the lumbar spine and femoral neck (Marques et al., 2012). It is important to consider, however, the inherent limitations of DXA that may lead to underestimation of the mechanical benefits of exercise (Petit et al., 2005). Based on attenuation of photons by bone and soft tissue, DXA provides a precise estimate of the amount of bone located within an area; however, it does not reveal bone structure (e.g., cross-sectional geometry, cortical thickness, microarchitecture).

Animal studies show changes in bone structure, as well as density, in response to exercise interventions, and these structural changes have major implications for bone strength (Robling et al., 2002; Warden et al., 2005). For example, loading of rat forelimbs increased both the strength of bone (+64%) and the energy required to fracture (+94%), while at the same time resulted in only a 7% increase in whole bone mineral content (Robling et al., 2002). This disproportionate increase in bone strength was attributed to deposition of bone on the periosteal surface, increased cross-sectional moment of inertia and, therefore, increased resistance to bending. Unfortunately, the majority of animal studies show age-related blunting of the periosteum's response to loading (Steinberg and Trueta, 1981; Raab et al., 1990; Rubin et al., 1992; Järvinen et al., 2003; Lieberman et al., 2003), with some studies reporting complete lack of response to exercise in older animals (Steinberg and Trueta, 1981; Rubin et al., 1992; Hoshi et al., 1998). In contrast, other studies indicate that mature and older animals retain the ability to mount a periosteal response (Chen et al., 1994; Leppanen et al., 2008). Exercise training resulted in equivalent gains in bone strength in 5- and 33-week-old rats (Järvinen et al., 2003); however, there were important qualitative differences in the structural alterations. The adult rats demonstrated increased volumetric bone mineral density (vBMD), whereas, the immature rats demonstrated greater periosteal apposition.

The disparate results of these prior studies may be due to differences in experimental design, including loading modality (treadmill running vs. functionally isolated ulnar loading) and bone measurement technologies (e.g., non-invasive imaging, mechanical testing, histomorphometry). Nevertheless, the overall indication from these studies is that bones in mature animals retain the ability to respond favorably to exercise, even if the response, particularly at the periosteum, is attenuated. These animal studies underscore the importance of obtaining structural as well as areal density information in human exercise studies.

Recent introduction of quantitative computed tomography (QCT), DXA-derived hip structural analysis (HSA), and magnetic resonance imaging (MRI) has afforded the ability to assess bone geometry, bone macro- and micro-structure, three-dimensional bone density, and estimates of bone strength using engineering analyses (Bouxsein, 2008). A systematic review of studies using either peripheral QCT (pQCT) or HSA to measure exercise effects on bone strength across various age groups reported improvements of 1–8% in children and adolescents, with either no change or very modest improvements in middle-aged and older adults (Nikander et al., 2010). Nevertheless, like DXA, HSA and pQCT have inherent limitations that make exercise studies difficult to interpret. HSA technology requires numerous assumptions to derive bone cross-sectional geometry from DXA scans, and pQCT lacks the resolution to capture the microstructural outcomes that have been demonstrated in animal exercise studies.

The ability to capture minute but mechanically significant changes in bone structure is a relatively recent development. High-resolution pQCT (HRpQCT) can detect changes in trabecular thickness, spacing, and number. It is not plagued to the same degree as standard pQCT with partial volume effects and should therefore, be more sensitive to changes in cortical thickness and periosteal and endosteal circumferences. Nevertheless, HRpQCT can only capture images in the distal appendicular skeleton. If exercise studies in adults and senescent individuals demonstrate favorable changes in the microarchitecture at the radius or tibia, assumptions will still have to be made about similar benefits occurring at clinically relevant sites such as the spine and hip, at least until high resolution imaging is available for these regions.

In summary, traditionally used imaging techniques that measure two-dimensional areal density (e.g., DXA) are not adequate to provide a comprehensive picture of exercise-induced changes in bone structure, and therefore, strength. Although, some animal studies employing mechanical testing and histomorphology demonstrate a positive effect of loading in adult and senescent animals, newer technologies (e.g., HRpQCT) will be necessary to fully reveal the beneficial effects of exercise on bone health in humans.

## Can non-traditional exercise regimens circumvent the need for high-impact loading by “hacking” the osteogenic signal?

Animal studies have shown that for skeletal loading to be anabolic, it must be dynamic and relatively intense such that resultant strains (or deformation of bone tissue) are high in magnitude (Lanyon and Rubin, 1984; Rubin and Lanyon, 1984). Higher magnitude strains are likely needed to initiate an anabolic response in the old vs. the young (Turner et al., 1995). High intensity resistance training in elderly humans has been conducted safely and has resulted in improvements in vBMD in the tibia and radius (Liu-Ambrose et al., 2004) and reduced risk of falls (Liu-Ambrose et al., 2005); nevertheless, high magnitude loading in the elderly remains controversial because of inherent risk of injury. The development of practical, safe, and effective osteogenic exercise programs will require creative solutions based on physiology of bone mechanosensitivity and adaptation and an understanding of the mechanisms underlying age-related declines in responsiveness to loading (Robling et al., 2006; Bonewald and Johnson, 2008; Dallas et al., 2013).

Loss of mechanosensitivity may be caused, at least in part, by age-related declines in the number and function of osteocytes (Frost, 1960; Wong et al., 1985; Dunstan et al., 1990). Aging is also associated with declines in the number and function of osteoblasts and the ability of these cells to differentiate from multipotent mesenchymal stem cells (Tsuji et al., 1990; Quarto et al., 1995; Majors et al., 1997; Nishida et al., 1999; Muschler et al., 2001; Yeh et al., 2015). Reduced mechanosensitivity may also be related to onset of menopause in women. Estrogen has been shown to play a role in regulating osteocyte paracrine signaling following mechanical loading (Joldersma et al., 2001) as well as osteocyte apoptosis (Tomkinson et al., 1997, 1998).

Clearly, further mechanistic research is needed to fully elucidate why bone becomes less sensitive to exercise with age. In the meantime, innovative solutions to combat age-related declines in bone mechanoadaptation are necessary, e.g., “rest-inserted loading” (Gross et al., 2004; Babatunde et al., 2012). Several studies in mature animals have demonstrated that rest periods of seconds (Srinivasan et al., 2002), hours (Robling et al., 2001), and even weeks (Saxon et al., 2005) inserted between loading cycles and bouts increase bone mechanoadaptation, resulting in greater gains in bone strength compared with continuous loading. In addition, low-magnitude loading with rest periods inserted produced a periosteal response in aged rats that was equal to that produced by loading with twice the magnitude, but without rest insertion (Srinivasan et al., 2003). A potential mechanism for the effectiveness of short-term rest is cellular accommodation, in which mechanosensitive cells adjust cytoskeletal stiffness in response to fluid flow, thereby inhibiting release of important pro-osteoblastic signaling molecules such as nitric oxide and prostaglandins (Jaasma et al., 2007). This mechanism may also explain cellular “saturation” in which bone cells become less mechanically sensitive with repetitive loading and why a small number of loading cycles seems to have the same osteogenic potential as a larger number of cycles (Umemura et al., 1997). Taken together, these studies suggest that only a few low- to moderate-intensity loading cycles with rest-insertion may be an adequate stimulus for bone formation.

Another modality that may circumvent the need for high-impact loading is low-amplitude, high-frequency vibration, an intervention that has been shown to increase bone formation in animal models of post-menopausal, peri-implant, and congenital osteoporosis (Shi et al., 2010; Vanleene and Shefelbine, 2013; Liang et al., 2014). The results of clinical investigations in post-menopausal women, however, have been inconsistent. In some studies, whole body vibration had beneficial effects on bone (Rubin et al., 2004; Beck and Norling, 2010; Lai et al., 2013), whereas, in other investigations, no positive outcomes were observed (Russo et al., 2003; Iwamoto et al., 2005; Slatkovska et al., 2014; Santin-Medeiros et al., 2015; Xu et al., 2016). Subgroup analysis performed as part of a recent systematic review points to several reasons for the disparate results (Oliveira et al., 2016). In that analysis, vibration-induced increases in BMD were observed only when: a side-alternating, high-frequency, low amplitude vibration waveform was applied, when subjects were positioned with semi-flexed knees, and when subjects who concomitantly received medications or performed additional forms of training were excluded from analysis. While this modality holds at least some promise in mature and post-mature individuals, clinical use of whole body vibration is not justified until the optimal vibration parameters, training frequency and duration, subject body position, and overall safety of this intervention have been determined.

One clinical condition in which the negative effect of aging on bone health seems to be accelerated is disuse osteoporosis following spinal cord injury (Dolbow et al., 2013). In this population, transcutaneous electrical stimulation of denervated muscle is one relatively recent strategy that, when used to elicit cycling or rowing exercise, has positive effects on bone. In case studies (Okafor et al., 2016), retrospective correlational analyses (Hammond et al., 2014), and randomized controlled trials reviewed by Chang et al. (2013), functional electrical stimulation has been shown not only to prevent bone loss, but to increase bone density and quality with seemingly greater effectiveness than traditionally employed interventions such as bisphosphonate administration, assisted standing, and weight supported treadmill walking (Lichy and Groah, 2012; Newman and Barker, 2012; Tan et al., 2013).

A final innovative strategy, non-customary loading, is based on the finding that abnormal loading patterns are more osteogenic than customary ones (Rubin and Lanyon, 1984). A likely explanation is that regions of bone experiencing the greatest strains also experience the greatest increases in bone formation (Cresswell et al., 2016). Bones already adapted to customary loads may require novel, multiaxial activities to initiate adaptive responses via greater local strains. A practical example might be a habitual walker who subjects her bones to “non-customary” loading patterns via ballroom dance (e.g., rapid changes in tempo and direction of movement, jumps, and weight shifts).

To summarize, animal, and human studies demonstrate that mature and senescent bone retains the ability to respond to loading with osteogenesis; however, innovative paradigms such as rest insertion and “non-customary” loading may be necessary to maximize the osteogenic potential of exercise.

## Can the physiologic interaction between bone and the sympathetic nervous system be exploited to optimize exercise-induced changes in bone health?

In the past, bone was viewed as a “calcium reservoir” and a system of scaffolding/support for other tissues. We now know that bone tissue interacts constantly with surrounding tissue, circulating factors, and other organ systems. Indeed, the force/tension relationships between skeletal muscle and bone are a major factor in the beneficial influence of exercise training on bone strength. Furthermore, circulating hormones have important influences on bone structure and function that have been recognized for decades. These hormonal effects are discussed extensively in recent reviews (Dallas et al., 2013; Girgis et al., 2015; Silva and Bilezikian, 2015; Taraborrelli, 2015). In this section, we highlight an emerging area of research—the relationship between bone and the sympathetic nervous system.

The proximity of sympathetic nerve endings to osteoblasts provides the anatomical substrate for this osteo-neural interaction. Evidence from animal models and clinical studies suggests that a β2-adrenergic receptor mechanism underlies the interaction (Elefteriou et al., 2005). In adult rats, chronic administration of β2-adrenergic agonists negatively affected bone density, microarchitecture, and mechanical properties (Bonnet et al., 2005). In addition, the clinical use of β-adrenergic antagonists is often (but not always Reid et al., 2005) associated with increases in trabecular density and/or decreases in bone turnover and risk of fractures in older people (Schlienger et al., 2004; Wiens et al., 2006). Age-related increases in sympathetic activity are well-documented in healthy humans (Dinenno and Joyner, 2006; Hart and Charkoudian, 2014); therefore, a β-adrenergic mechanism may contribute to decreases in trabecular microstructure that occur with normal aging.

This question was recently addressed in pre- and post-menopausal women using direct measurements of sympathetic neural activity along with DXA measures of areal BMD and HRpQCT measures of cortical and trabecular bone microstructure and volumetric BMD (Farr et al., 2012). In this study, higher sympathetic activity was associated with lower trabecular number and connectivity density. Interestingly, these negative relationships were stronger in the distal radius vs. the tibia, suggesting that load-bearing activities (i.e., many forms of exercise), may blunt, or minimize any deleterious influence of sympathetic activity on bone structure or strength. Plasma levels of osteopontin, a bone regulating factor that contributes importantly to the negative influence of β-adrenergic agonists on bone tissue (Nagao et al., 2011), were also inversely related to sympathetic activity in younger and older women. This finding suggests the possibility of a negative feedback loop that would minimize the negative influences of β-adrenergic stimulation on bone metabolism in individuals with elevated sympathetic nerve activity.

Studies in adult mice and rats indicate that inactivity or disuse results in bone loss (Cabahug-Zuckerman et al., 2016) and increased sympathetic nervous system activation (Mischel and Mueller, 2011). Inhibition of disuse-associated elevation in sympathetic tone using a β-blocker mitigated bone loss by altering osteoblast and osteoclast activity (Kondo et al., 2005; Baek and Bloomfield, 2009). In contrast to disuse, exercise training in animal models reduces sympathetic activity (see Mueller, 2007 for review). The beneficial effect of exercise training on bone formation in adult rats was prevented with a β2-adrenergic agonist (Bonnet et al., 2007), suggesting that the negative effects of sympathetic stimulation can override the positive effects of load-bearing exercise training on bone formation.

Recent observational studies of patients with spinal cord injury indicate that bone mineral content is more compromised in patients with paraplegia than those with quadriplegia (Coupaud et al., 2009; Dionyssiotis et al., 2009). This effect is likely attributable to the smaller muscle mass and the flaccid vs. spastic paralysis characteristic of low- vs. high-level lesions; however, it is tempting to speculate that at least some of this effect could be explained by comparatively more complete sympathetic innervation below the level of the lesion in individuals with paraplegia.

Mechanistic studies in adult mice indicate that the sympathetic nervous system is a key regulator of bone formation. In a murine model of immobilization stress, circulating levels of norepinephrine increase, and bone loss occurs. In contrast, after sympathectomy, norepinephrine decreases and the bone loss produced by immobilization is reversed (Jiao et al., 2015). Further supportive evidence comes from studies in which hypothalamic function was genetically altered by manipulation of Single minded 1 (Sim1), a transcription factor necessary for hypothalamic development. In a case study describing a patient with a Sim1 mutation, extremely high BMD was reported. In subsequent murine experiments, adult deletion of Sim1 increased bone formation by reducing sympathetic activity (Wang et al., 2015). The authors then confirmed the role of adrenergic activity by administering a β-adrenergic receptor agonist to Sim1 knockout mice, which reversed the increased bone formation associated with Sim1 deletion.

Thus, several lines of evidence demonstrate that sympathetic stimulation adversely affects integrity of the adult skeleton. Whether, similar mechanisms are responsible for the negative relationship between elevated sympathetic activity and low bone density in post-mature individuals (Farr et al., 2012) has not yet been tested.

Although, sympatholytic effects of exercise training have been observed in experimental animals, most human studies have found that sympathetic nerve activity is unaffected by training in young, healthy adults—individuals in whom baseline levels of sympathetic outflow are relatively low (Hart and Charkoudian, 2014; Carter and Ray, 2015). In contrast, exercise training produces rather dramatic decreases in sympathetic outflow in individuals with high basal nerve traffic (e.g., patients with heart failure (Roveda et al., 2003; Negrão and Middlekauff, 2008) and hypertension (Grassi et al., 1992; Laterza et al., 2007). The potential sympatholytic effects of exercise training in healthy older adults, who also have relatively high basal sympathetic activity, and the impact of exercise-induced reductions in sympathetic activity on bone health remain largely unexplored.

A potential integrative mechanism underlying the link between the sympathetic nervous system and bone is the vestibulo-sympathetic reflex. Evidence from microgravity studies, showing upregulated sympathetic nerve activity and concurrent accelerated bone loss, suggests that vestibular signals may be important in bone remodeling (Vignaux et al., 2013). Experimental manipulations of vestibular inputs in humans, i.e., long-term head-down bedrest (Tanaka et al., 2013) or space flight (Ertl et al., 2002), are also associated with increases in sympathetic outflow and decreases in bone mass (Ertl et al., 2002; Armbrecht et al., 2011; Tanaka et al., 2013; Smith et al., 2014). In the converse situation (i.e., increased gravitational stress), alterations in vestibular input may contribute to the link between upright, weight bearing locomotion, and beneficial bone remodeling.

In summary, studies in animals and humans suggest that the sympathetic nervous system may be an important mechanistic link between physical activity and bone health. This emerging evidence raises the possibility that pharmacological intervention may be able to augment the benefits of exercise on bone health in humans across the lifespan.

## Conclusions

**Figure 1:**
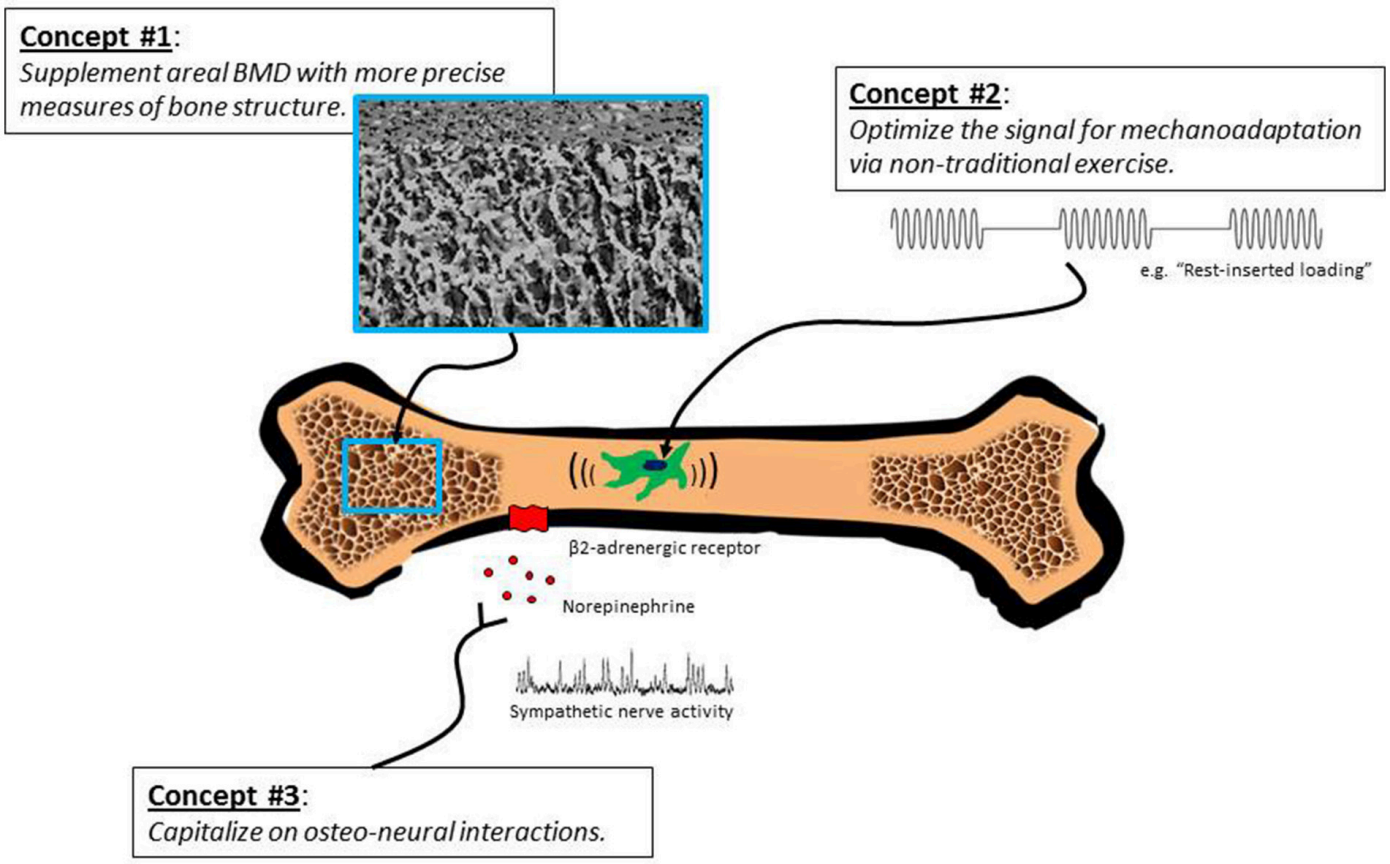
**Several sometimes overlooked concepts call into question the notion that exercise interventions are minimally effective in building bone strength in older adults**. We propose that exercise can produce favorable changes in architecture and strength of mature and post-mature bone; however, revelation of this capability may require innovative exercise programs, high-resolution imaging techniques, and interventions that capitalize on bone-sympathetic nervous system interactions.

Multiple lines of evidence from human and animal studies indicate that the aging skeleton remains modestly responsive to exercise interventions. In Figure 1, we outline an approach for further documentation of these effects. Retained responsiveness of bones to exercise may require more intense loading stimuli in older compared with younger individuals; nevertheless, unique strategies for “hacking” the system (e.g., rest-inserted loading, non-customary loading, vibration) can possibly obviate the need for extreme loads. Pharmacological interventions that take advantage of sympathetic neural-bone interactions (e.g., β-adrenergic blockade) may be another potentially fruitful strategy. Sophisticated imaging methods are needed to fully characterize exercise-induced adaptations in bone architecture and bone strength in older populations. We propose that exercise has the potential to positively affect bone health in mature and elderly individuals, and regardless of the degree to which exercise improves bone strength, prevention of bone loss is, *per se*, a desirable outcome in this population.

## Author contributions

All authors listed, have made substantial, direct and intellectual contribution to the work, and approved it for publication.

## Funding

This work was supported by grant #HL118154 to JB from the National Heart, Lung, and Blood Institute, United States.

## Disclaimer

The opinions or assertions contained herein are the private views of the authors and are not to be construed as official or reflecting the views of the U.S. Army or the Department of Defense.

### Conflict of interest statement

The authors declare that the research was conducted in the absence of any commercial or financial relationships that could be construed as a potential conflict of interest.
